# A statistical test and sample size recommendations for comparing community composition following PCA

**DOI:** 10.1371/journal.pone.0206033

**Published:** 2018-10-24

**Authors:** John R. Skalski, Shelby M. Richins, Richard L. Townsend

**Affiliations:** School of Aquatic & Fishery Sciences, University of Washington, Seattle, Washington, United States of America; University of Illinois at Urbana-Champaign, UNITED STATES

## Abstract

Many investigations of anthropogenic and natural impacts in ecological systems attempt to detect differences in ecological variables or community composition. Frequently, ordination procedures such as principal components analysis (PCA) or canonical correspondence analysis (CCA) are used to simplify such complex data sets into a set of primary factors that express the variation across the original variables. Scatterplots of the first and second principal components are then used to visually inspect for differences in community composition between treatment groups. We present a multidimensional extension of analysis of variance based on an analysis of distance (ANODIS) that can be used to formally test for differences in community composition using 1, 2, or more dimensions of a PCA or CCA of the original sample observations. The statistical tests of significance are based on *F-*statistics adapted for the analysis of this multidimensional data. Because the analysis is parametric, power and sample size calculations useful in the design of field studies can be readily computed. The use of ANODIS is illustrated using bivariate PCA scatterplots from three published studies. Statistical power calculations using the noncentral *F-*distribution are illustrated.

## Introduction

Multivariate analysis is often exploratory or descriptive rather than inferential in nature. One reason for the preponderance of descriptive studies is that investigators often collect unwieldy amounts of data per sample in an attempt to uncover important patterns. Overparameterization of the information then forces investigators to use *post hoc* methods such as cluster analysis, principal components analysis (PCA), or canonical correspondence analysis (CCA) to summarize and simplify the sample comparisons. Ordination procedures such as PCA offer a systematic way to reduce dimensionality of complex data sets and organize it into new independent composite variables (i.e., the principal components). Thus, redundancy in the data set is minimized and sampling entities are organized around a few important gradients, which aid descriptive interpretation [1–3]. In a sense, it eliminates concern over bias due to linearly correlated variables by providing the best signal-to-noise ratio for separating differences in structures.

Many ecological studies that seek to detect changes in community composition or differences in habitat structure are hampered by a lack of formal statistical methods to test for differences in principal components. Currently, investigations of anthropogenic and natural impacts on biological communities use many different analytical methods to assess effects. For example, following the *Exxon Valdez* oil spill in Prince William Sound, AK, investigators [4–6] resorted to multiple species-by-species *t*-test analyses, adjusting the test-wise α-levels for the multiple tests to control the experimental-wise Type I error rate. In another *Exxon Valdez* study, Day et al. [7] used separate one-way ANOVAs on the first two principal components. Separate one-way ANOVAs on principal components were also used by Towns et al. [8], who sought to investigate the impact of invasive rats on invertebrate community composition in island soils. More often, however, researchers simply provide qualitative descriptions of community patterns based on bivariate plots of principal component values. In these instances, PCA bivariate scatterplots of the first two principal components may suggest community patterns or gradients, but objective interpretation is often difficult [9–15] and statistical significance is not mentioned.

Because visual inspection of PCA bivariate scatterplots is sensitive to personal perspective, objective approaches to data interpretation are necessary. Without objective criteria for comparison of PCA scatterplot data, it is also difficult to determine required sample sizes. Krzanowski [16] offers some guidance on sample sizes when comparing principal components between two treatment groups. However, the guidance is of limited value because it pertains to a few specific values of the “percent trace in the first population component.” Otherwise, Monte Carlo methods must be used to generalize the approach. Saccenti and Timmerman [17] provide guidance on minimal sample sizes for principal component comparison but without considering Type I and Type II statistical error rates. In the absence of convenient statistical guidance, sample sizes are often driven by intuition, personal preference, or budgetary constraints rather than *a priori* study performance. Inadequate sample sizes for performing community composition comparisons further contribute to the subjective interpretation of the data. Without rigorous design, studies are often relegated to simply qualitative impressions. For these many reasons, ecologists are often left in a quandary over what statistical analyses are appropriate to evaluate even the most basic hypotheses concerning differences in community composition following PCA [18].

We present a multidimensional extension of analysis of variance based on an analysis of distance (ANODIS) that can be used to test for differences in community composition using 1, 2, or more dimensions of a PCA or CCA of the original sample observations. The tests of significance are based on *F-*statistics adapted specifically for the analysis of these multidimensional data. These statistical tests can be generalized to a variety of experimental designs, can simultaneously analyze multiple dimensions of a principal components analysis, and their noncentral distributions can be used in the design of experiments. A parametric approach to data analysis was chosen because it readily permits power and sample size calculations useful in the design of field studies. We believe proper design of studies is as important as proper data analysis, so both the design and analysis aspects of PCA bivariate scatterplot data are presented.

## PCA bivariate scatterplot examples

The ANODIS methods will be illustrated using published PCA bivariate scatterplot data from three different studies (Magoba and Samways [11], Louys et al. [13], and Annala et al. [14]). The PCA values (S1–S3 Files) were digitally extracted from printed figures in the previously published studies using GetData Graph Digitizer 2.26 and, as such, may not exactly represent the original values. The subsequent analyses of the digitized PCA values using ANODIS should therefore be considered for the purpose of illustration only.

### Dragonfly assemblages [11]

**Fig 1:**
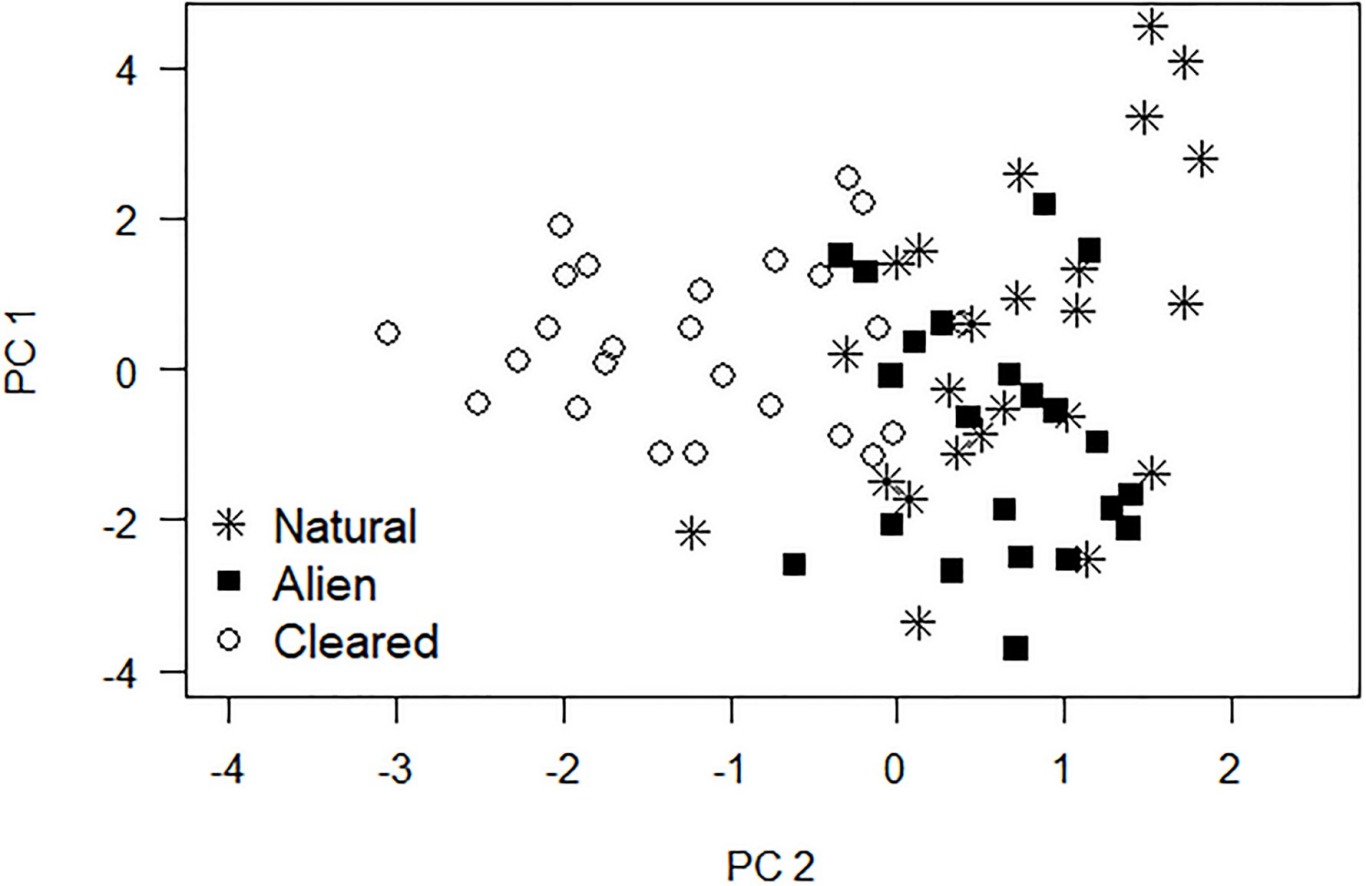
Bivariate scatterplot of dragonfly count data. A scatterplot based on the first two principal components of hypergeometric probabilities derived from dragonfly count data collected in riparian zones in South Africa. This figure is based on data digitally extracted from Magoba and Samways (p. 632, fig. 4 in [11]) and is therefore for illustrative purposes only. Riparian zones included natural trees, a mix of natural and alien trees, or had alien trees cleared.

Following the removal of hundreds of hectacres of invasive trees in South Africa, Magoba and Samways [11] examined differences in adult dragonfly community composition between riparian zones with contrasting vegetation types. Three headwater streams with minimal anthropogenic impacts were selected from the Luvuvhu River basin. Dragonfly assemblages were examined in three riparian zone classes: (1) zones with only natural vegetation, (2) zones with dense alien vegetation, and (3) zones manually cleared of alien vegetation. Twenty-three 10 x 2 m stream stretches were drawn from natural and alien stream segments, and twenty-five stretches were taken from areas cleared of alien vegetation. In 30-minute sampling periods, adult dragonflies were visually identified to species. Because female dragonflies are rarely associated with water, only male dragonflies were recorded. Voucher specimens were collected with butterfly nets to confirm species identifications. To compare differences in dragonfly community composition, Magoba and Samways [11] created a bivariate scatterplot of the first two principal components derived from Bray-Curtis similarity measures (Fig 1).

### Mammalian assemblages [13]

**Fig 2:**
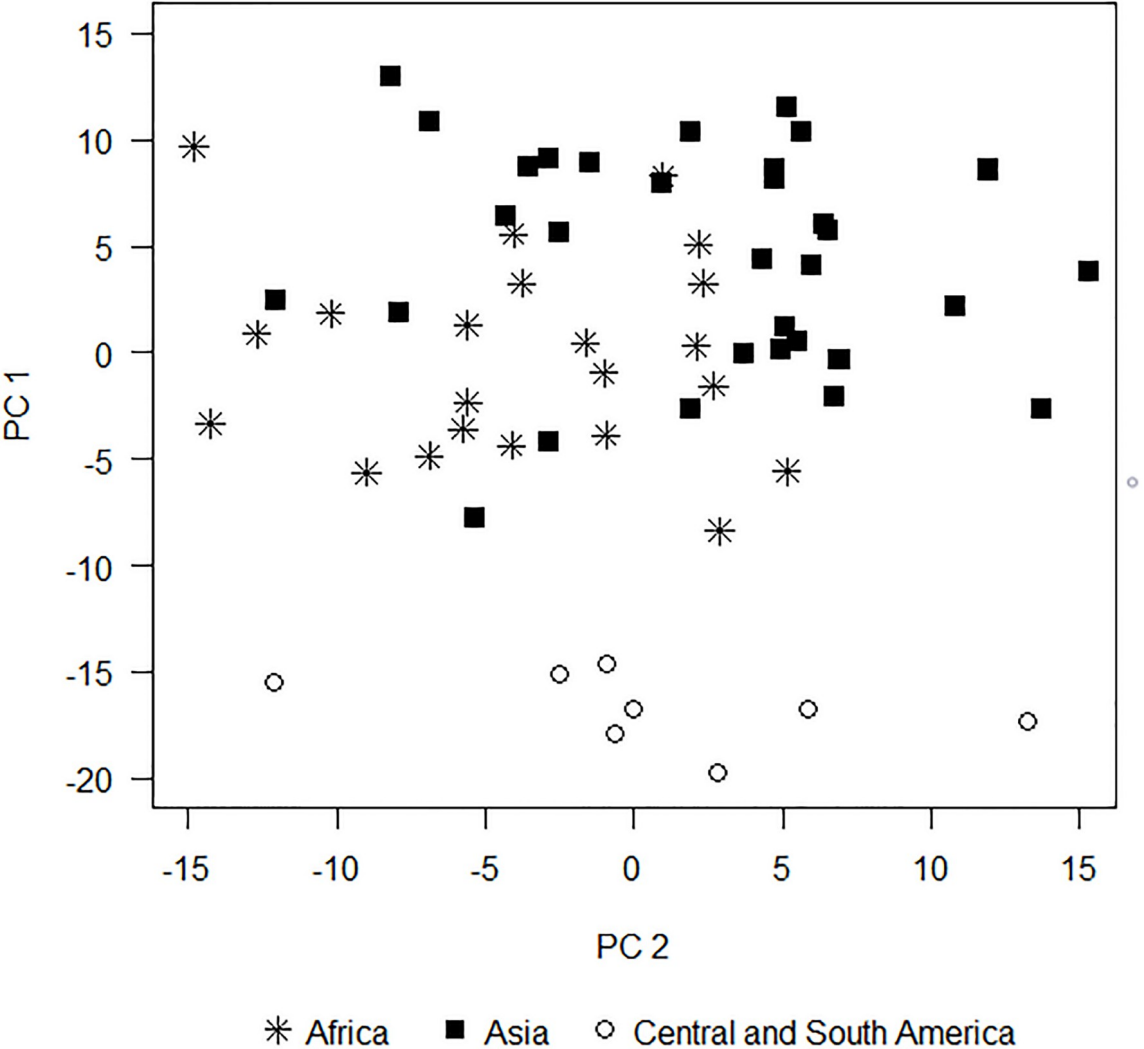
Bivariate scatterplot of mammalian community structure. A scatterplot based on the first two principal components of hypergeometric probabilities derived from mammal community structure in Asia, Africa, and South and Central America. This figure is based on data digitally extracted from Louys et al. (p. 726, fig. 5 in [5]) and is therefore for illustrative purposes only. The authors first corrected for habitat to examine the influence of continental history on mammal community structure.

To understand the influence of historical effects on mammal community composition, Louys et al. [13] examined mammalian assemblages in natural protected areas across three continents: Asia, Africa, and Central/South America. Using the Man and the Biosphere Species Database (http://ice.ucdavis.edu/mab), species presence/absence were recorded for 23 African, 32 Asian, and 8 Central and South American natural protected areas. Species were categorized by mass (<1 kg, 1–10 kg, 10–45 kg, 45–180 kg, and >180 kg), trophic level (primary or secondary consumer), and locomotion (terrestrial or arboreal). To first correct for variation due to environmental factors, percent heavy tree cover was calculated based on satellite imagery obtained from Google Earth (see Louys et al. [13]). Ecological guilds were regressed against tree cover and principal components analysis was carried out on the residuals. The results of their PCA were illustrated using a bivariate scatterplot (Fig 2).

### Stream chemistry [14]

**Fig 3:**
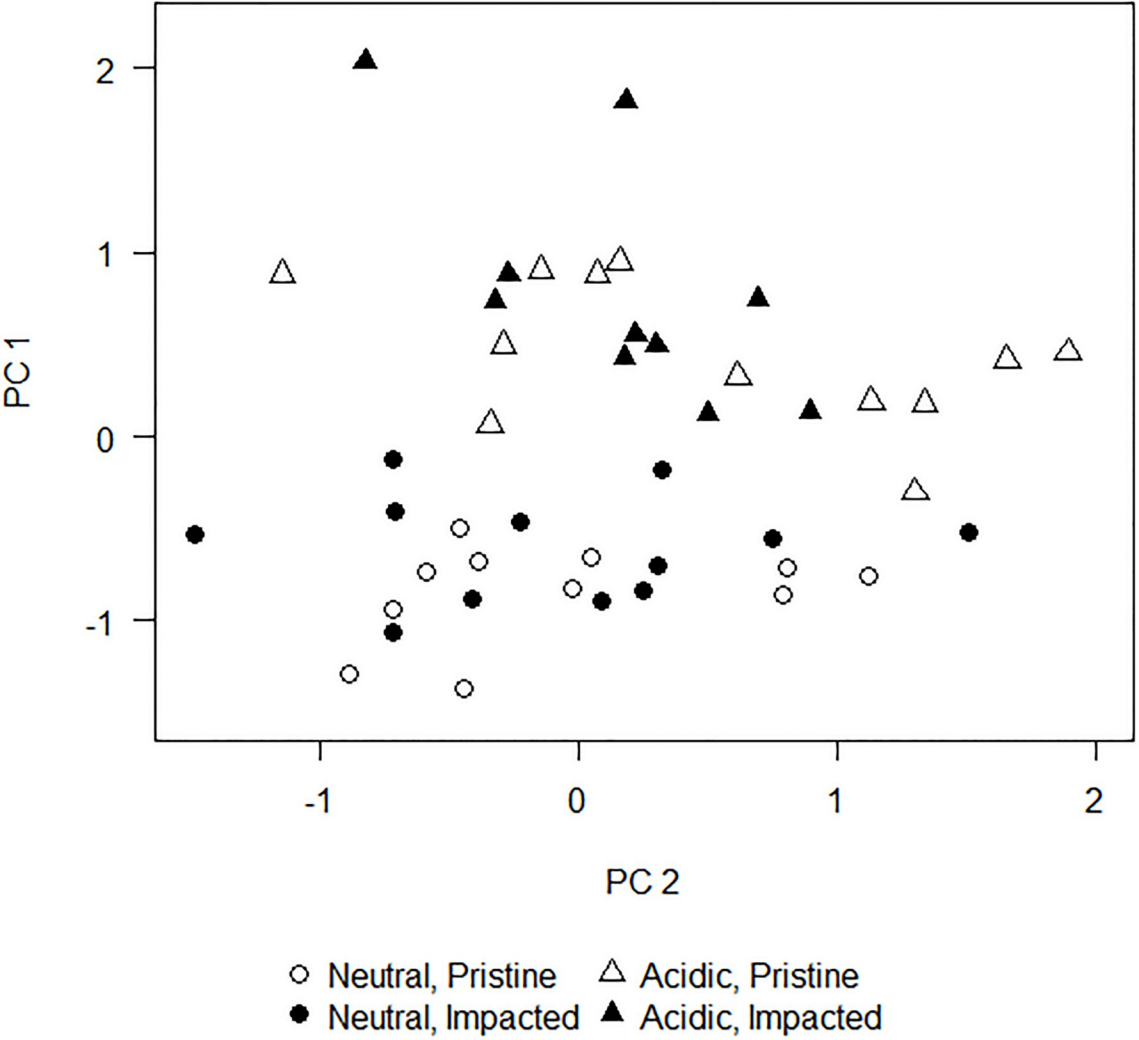
Bivariate scatterplot of stream chemistry. A scatterplot based on the first two principal components derived from water chemistry data collected in streams in northern Finland with different combinations of stressors. This figure is based on data digitally extracted from Annala et al. (p. 1891, fig. 1B in [14]) and is therefore for illustrative purposes only. Neutral = no acidic stress, Acidic = acidic stress resulting from underlying geology, Pristine = no land use impacts, Impacted = land use impacts, primarily drainage ditching for forestry activities.

To determine whether naturally stressed streams may be resistant to further anthropogenic stress, Annala et al. [14] sampled water chemistry variables in streams in northern Finland, including 24 in the naturally acidic Iijoki basin and 24 in the circumneutral Oulujoki basin. The naturally acidic streams ran over black shale deposits and had an average summer pH of 5.0 (range 3.7–5.9), while the circumneutral streams had an average summer pH of 6.5 (range 5.4–7.2). In both basins, drainage ditching as part of forestry activities was the primary anthropogenic impact. In each basin, 10 to 12 sites were samples in the impacted and near-pristine treatment groups. All sampling occurred in riffle sections in headwater streams. Water samples were collected in October 2010, following the methods used by the Geological Survey of Finland [19]. Samples were analyzed for electrical conductivity, alkalinity, pH, total phospohorus, dissolved organic carbon, sulphate (SO_4_), and metals (Cu, Mn, Ni, Pb, and Zn) in the laboratory. Principal components analysis was then used to summarize differences in overall water chemistry between streams (Fig 3). To formally test for natural and anthropogenic effects on water chemistry, we used ANODIS. The variance was partitioned among the main effects of stream geology and impact status, as well as the interaction between the two main effects.

## Statistical methods

### Analysis of distance

By design, principal components generated by PCA are orthogonal [20, 21]. In other words, second and higher order axes are independent of all previous axes. The property of independence allows the analysis of the separate components to be combined based on the principle that the sum of independent chi-square statistics is also chi-square distributed with degrees of freedom equal to the sum of the separate degrees of freedom.

The distance (*D*) between two points (*x*_1_, *y*_1_) and (*x*_2_, *y*_2_) in Cartesian space is calculated as

$$D=\sqrt{{\left(x_{1}-x_{2}\right)}^{2}+{\left(y_{1}-y_{2}\right)}^{2}}.$$

Consequently, the distance squared (*D*^2^) between the two points is the sum of the separate squares for the *x*-coordinates and the *y*-coordinates, where

$$D^{2}={\left(x_{1}-x_{2}\right)}^{2}+{\left(y_{1}-y_{2}\right)}^{2}.$$ (1)

In our application, the *x* and *y* coordinates correspond to the first and second principal components of a bivariate scatterplot.

**Fig 4:**
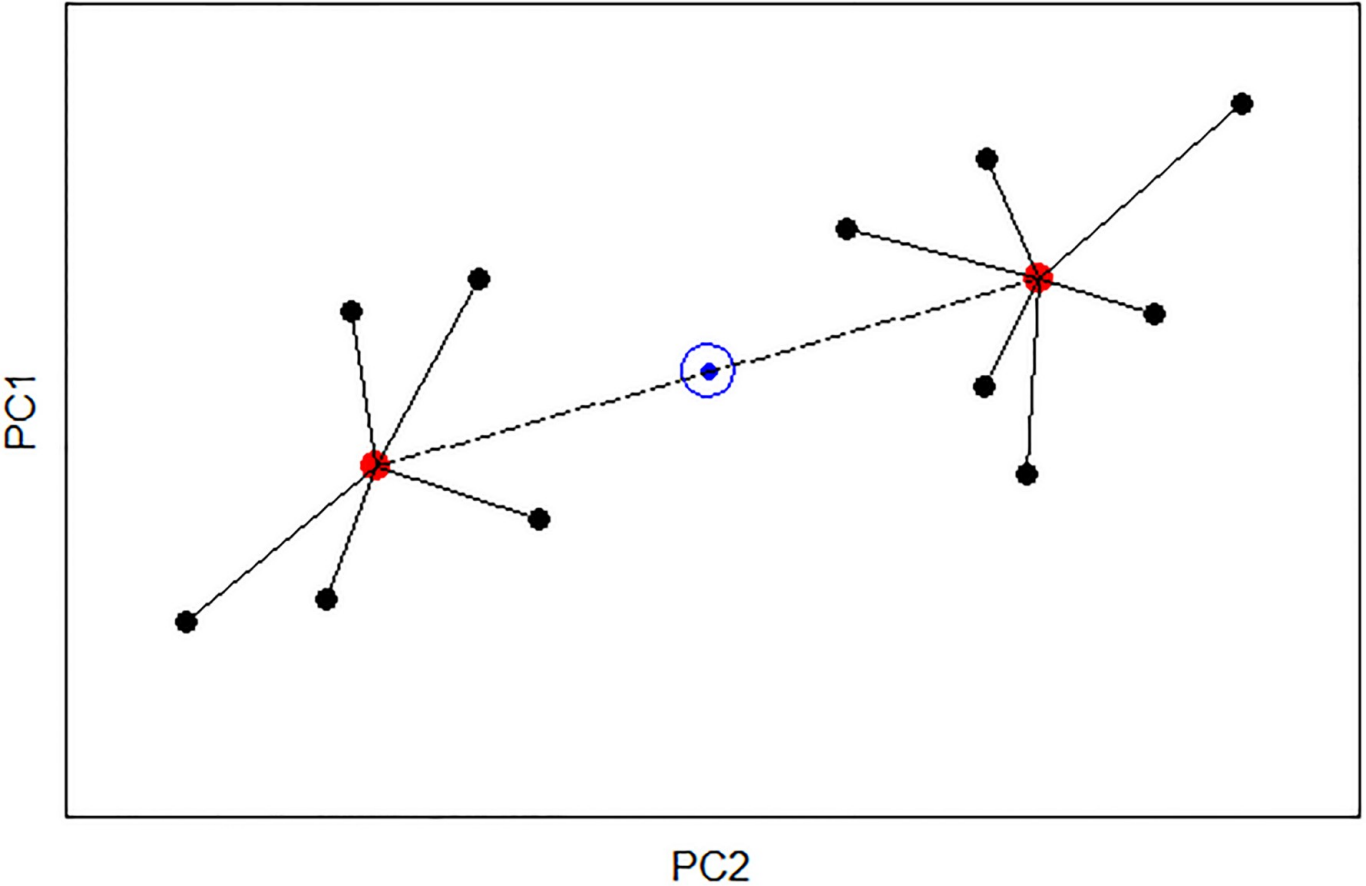
Illustration of PCA bivariate scatterplot. Scatterplot of two principal components from two communities with replicate samples, along with within-group (red dot) and between-group (blue dot in circle) centroids.

Consider the situation where independent samples were collected from different communities one wishes to compare using the results from PCA. Geometrically, the ANODIS partitions the total sum of squared distance from the centroid of the combined data into between-treatment and within-treatment components (Fig 4 and S1 Appendix). This partitioning can be performed on the separate *x* and *y* coordinates and then recombined as per Eq (1). In the dragonfly bivariate PCA scatterplot example (Fig 1), three treatment groups are being compared visually. In general, any ANOVA that can be constructed to analyze univariate data has an ANODIS analog when analyzing principal components. In particular, the ANODIS for any univariate data set is the ANOVA. The null hypothesis (H_o_) for the dragonfly bivariate scatterplot (Fig 1) is that the samples are from a single population with common centroid equivalent to the bivariate mean (*μ*_*x*_, *μ*_*y*_). The alternative hypothesis is that the samples are from three different populations with centroids $\left(\mu_{x_{i}},\;\mu_{y_{i}}\right)$ for *i* = 1, 2, 3.

The ANODIS can be easily constructed by combining the results of the separate ANOVAs for each of the principal components analyzed (Table 1, and R code example in the S4 File). While Table 1 shows the construction of an ANODIS table for two dimensions, the extension to three or more dimensions is straightforward. The degrees of freedom for the resulting *F-*test in ANODIS will be increased over that of ANOVA by a factor equivalent to the number of principal component dimensions analyzed (Table 1, S1 Appendix). Statistical significance and associated *P-*values can be read from standard *F-*tables.

**Table 1:** An example of combining ANOVA terms for bivariate principle component data to create the ANODIS *F-*statistic where *N* is the total number of samples drawn and *K*, the number of assemblages compared.

Source	*PC1- ANOVA*		*PC2-ANOVA*		ANODIS	
	DF	SS	DF	SS	DF	SS
**Total corrected**	*N* − 1	SS_Tot.x_	*N* − 1	SS_Tot.y_	2(*N* − 1)	SS_Tot.x_ + SS_Tot.y_
**Between treatment**	*K* − 1	SS_Treat.x_	*K* − 1	SS_Treat.y_	2(*K* − 1)	SS_Treat.x_ + SS_Treat.y_
**Within error**	*N* − *K*	SS_Error.x_	*N* − *K*	SS_Error.y_	2(*N* − *K*)	SS_Error.x_ + SS_Error.y_
***F-*statistic**	$\frac{\left(\frac{{SS}_{Treat.x}}{\left(K-1\right)}\right)}{\left(\frac{{SS}_{Error.x}}{\left(N-K\right)}\right)}$		$\frac{\left(\frac{{SS}_{Treat.y}}{\left(K-1\right)}\right)}{\left(\frac{{SS}_{Error.y}}{\left(N-K\right)}\right)}$		$\frac{\left[\frac{{SS}_{Treat.x}+{SS}_{Treat.y}}{2(K-1)}\right]}{\left[\frac{{SS}_{Error.x}+{SS}_{Error.y}}{2(N-K)}\right]}$	

**Fig 5:**
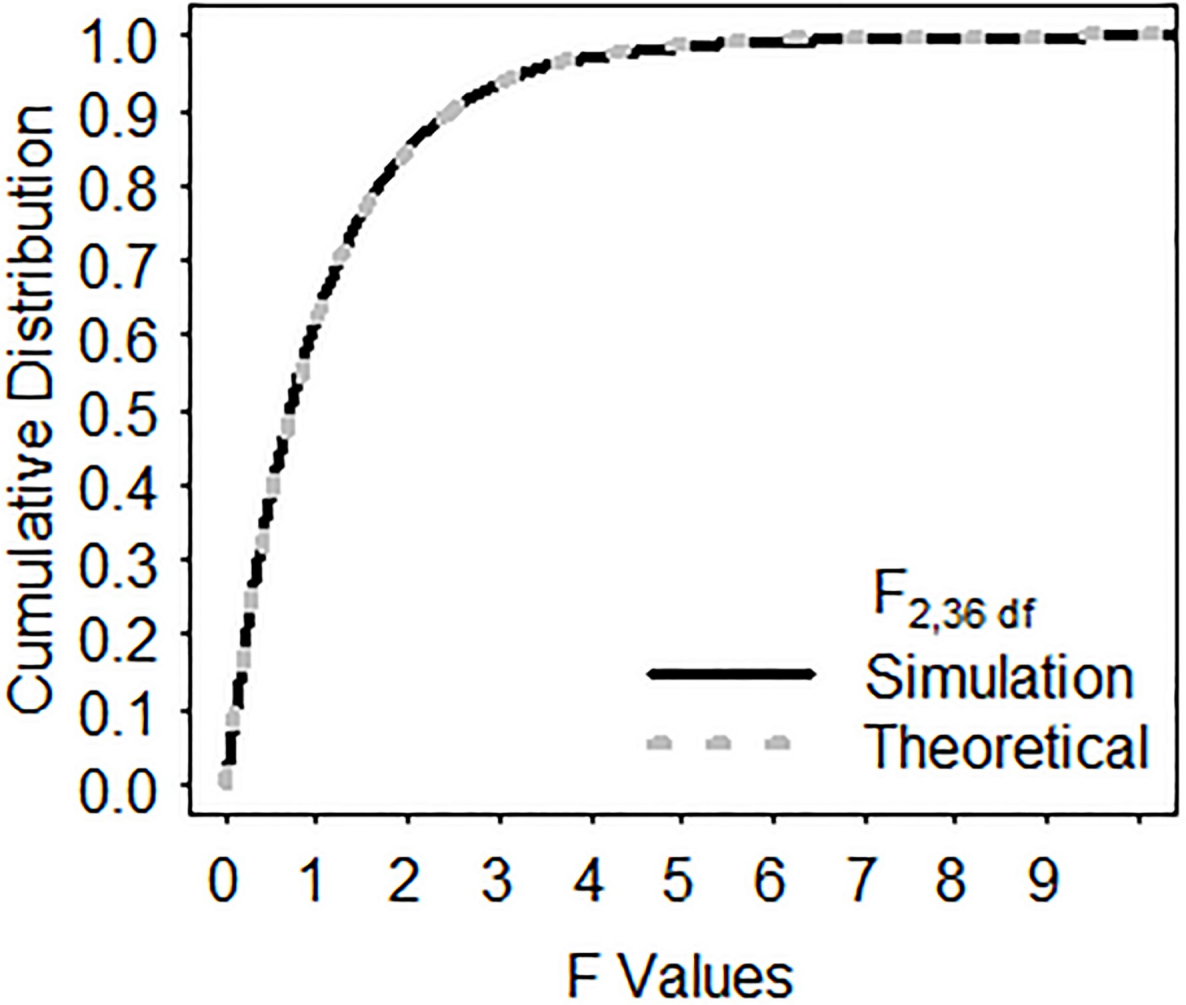
Example of simulation results under H_0_. Comparison of the empirical cumulative distribution of ANODIS *F*-test with the theoretical *F*-distribution with *d*_1_ = 2 and *d*_2_ = 36 degrees of freedom for the case of *K* = 2 communities, *r* = 5 species per community assemblage, and *n* = 10 samples per community under the null hypothesis of homogeneous centroids. Other simulated scenarios produced similar results of perfect fit of simulated and expected *F*-distributions.

To confirm the distribution theory leading to the *F-*test for ANODIS, we performed Monte Carlo simulations. We generated vectors of community abundance based on an *r*-dimensional multivariate normal distribution. Simulations varied the number of dimensions (*r* = 5, 7, or 10), the mean abundance levels, and the variance–covariance relationships between the simulated populations within a community. Varying numbers of samples per community assemblage, *n* = 5, 10, 15, or 20 were drawn under the null hypothesis of a common assemblage, and PCA was used to calculate the first two principal components across the total samples drawn from a *K* treatment comparison (i.e., $N=\sum_{i=1}^{K}n_{i}$). Scenarios of unequal sample sizes were also simulated. Simulations examined scenarios with *K* = 2, 3, or 4 community assemblages being compared simultaneously. For each scenario simulated, 10,000 replicate Monte Carlo simulations of the study were generated. For each replicate, the *F*-test for the ANODIS was computed, and their empirical cumulative distribution compared to the cumulative distribution function (cdf) of an *F*-statistic with 2(*K*– 1)and 2(*N*–*K*) degrees of freedom. The empirical cumulative distributions for the generated *F-*tests agreed exactly with the cdf of the theoretical *F*-distributions in all scenarios simulated (Fig 5).

For each of the bivariate principal component scatterplot examples drawn from the literature, we also performed one-way ANOVAs (i.e., linear model ln (*y* ~ factor(TREATMENT), data = data.name) in R) on the separate principal components, and used Hotelling’s *T*^2^ (i.e., the function “hotelling.test” in the Hotelling package in R) to compare assemblages. These example analyses were used to empirically compare the ANODIS results to more traditional methods.

In addition, Monte Carlo simulations were performed to compare the statistical power (i.e. 1-β) of these three alternative methods of data analysis. Power curves were generated for each method as a function of the size of the distance between community centroids at α = 0.05, 2-tailed. The scenario’s simulated under the alternative hypothesis (i.e. Ha) consisted of the same range in r, n and K values as performed under the null (i.e. Ho). In the case of multiple community comparisons under Ha, minimal power scenario was simulated where 1 community differed from the remaining K-1 communities. The test-wise *α*_*TW*_−level of the individual tests in the case of separate ANOVAs for the different principle components was adjusted by the formula 1 –(1 − *α*_*tw*_)^2^ = 0.05 or α = 0.0253 to maintain the same overall experimental-wise error rate *α*_*ex*_ = 0.05 as ANODIS and Hotelling’s T^2^.

#### Statistical power and sample size determination

An advantage of the *F-*test derived from the ANODIS is that the distributional properties of the test statistic are well described under both null and alternative hypotheses because of the ANOVA underpinnings. Noncentral *F-*distributions can be used to determine the statistical power of tests and perform sample size calculations. No additional statistical theory needs to be developed, and existing software in the R statistics package can be directly applied to the question of required sample sizes for community composition studies. Statistical power for the *F*-test of equal centroids (Table 1) can be based on its noncentral distribution under the alternative hypothesis of differences in treatment locations. The noncentral *F-*distribution depends on the degrees of freedom in the numerator (df_1_) and denominator of the *F-*test (df_2_), and a noncentrality parameter.

Starting with the definition of the noncentrality parameter used by R for univariate analyses (denoted by $\lambda_{{df}_{1},{df}_{2}}$) for a noncentral *F*-distribution

$$\lambda_{{df}_{1}{,df}_{2}}=\frac{\sum_{i=1}^{K}n_{i}{\left(\mu_{i}-\overset{-}{\mu }\right)}^{2}}{\sigma^{2}}$$ (2)

where the quantity ${\left(\mu_{i}-\overset{-}{\mu }\right)}^{2}$ is the squared distance between the *i*th mean (*μ*_*i*_) and the grand mean $\left(\overset{-}{\mu }\right)$ across *K* treatment groups in 1-dimensional space. The quantity *σ*^2^ is the between-replicate, within-treatment variance defined as

$$\sigma^{2}=\frac{\sum_{i=1\;}^{K}{\sum_{j=1}^{n_{i}}\left(x_{ij}-\;\mu_{i}\right)}^{2}}{\sum_{i=1\;}^{K}\left(n_{i}-2\right)}$$

and where

*x*_*ij*_ = *j*th observation (*j* = 1, …,*n*_*i*_) in the *i*th treatment group (*i* = 1,…,K),

*n*_*i*_ = sample size for the *i*th treatment group (*i* = 1,…,K).

In the case of 2-dimensional space, λ can be rewritten where the distance now between treatment group centroids and the grand centroid can be expressed as

$$\lambda =\frac{\sum_{i=1}^{K}n_{i}({\left(\mu_{x_{i}}-{\overset{-}{\mu }}_{x}\right)}^{2}+{\left(\mu_{y_{i}}-{\overset{-}{\mu }}_{y}\right)}^{2})}{\sigma^{2}}$$

where x and y now denote the first and second orthogonal principal components and where $\mu_{x_{i}}$ (*i* = 1,…, K) is the mean of PC1 for the *i*th treatment, ${\overset{-}{\mu }}_{x}$ the grand mean for PC1 and $\mu_{y_{i}}$ and ${\overset{-}{\mu }}_{y}$ defined analogously. The quantity *σ*^2^ is now the between-replicate, within-treatment variance for the distances between individual replicate observations (*x*_*ij*_, *y*_*ij*_) and their treatment specific centroids (${\overset{-}{\mu }}_{x_{i}},\;{\overset{-}{\mu }}_{y_{i}}$) such that

$$\sigma^{2}=\frac{\;\sum_{i=1}^{K}\sum_{j=1}^{n_{i}}\left[{\left(x_{ij\;}-\;\mu_{x_{i}}\right)}^{2}\;+\;{\left(y_{ij}-\;\mu_{y_{i}}\right)}^{2}\right]}{\sum_{i=1}^{K}(n_{i}-2)}$$

In the case of two treatments (i.e. K = 2) and equal sample sizes (*n*_1_ = *n*_2_ = *n*), λ reduces to

$$\lambda_{2,\;2(n-2)}=\frac{n\sum_{i=1\;}^{2}{(\mu }_{x_{i}}\;-\;{\overset{-}{\mu }}_{x})+\;n\sum_{i=1}^{2}(\mu_{y_{i}}-\;{\overset{-}{\mu }}_{y}{)}^{2}}{\sigma^{2}}$$

where

$$\sigma^{2}=\frac{\sum_{i=1}^{2}\sum_{j=1}^{n}\left[{\left(x_{ij}-\;\mu_{x_{i}}\right)}^{2}+\;\sum_{i=1}^{2}\sum_{j=1}^{n}(y_{ij}-\;\mu_{y_{i}}\;{)}^{2}\right]}{2(n-2)}.$$

In this special case of K = 2

$$\sum_{i=1}^{2}(\mu_{x_{i}}-\;{\overset{-}{\mu }}_{x}{)}^{2}=\frac{(\mu_{x_{1}}-\;\mu_{x_{2}}{)}^{2}}{2}$$

and similarly

$$\sum_{i=1}^{2}(\mu_{y_{i}}-\;{\overset{-}{\mu }}_{y}{)}^{2}=\frac{(\mu_{y_{1}}-\;\mu_{y_{2}}{)}^{2}}{2}.$$

It then follows that

$$\lambda_{2,\;2(n-2)}=\frac{n((\mu_{x_{1}}-\;\mu_{x_{2}}{)}^{2}\;-\;(\mu_{y_{1}}-\;\mu_{y_{2}}{)}^{2})}{2\sigma^{2}}.$$

where

$$\sigma^{2\;}=\frac{\sum_{i=1}^{2}\sum_{j=1}^{n}{\left(x_{ij}-\;\mu_{y_{i}}\right)}^{2}+\sum_{i=1}^{2}\sum_{j=1}^{n}{\left(y_{ij}-\;\mu_{y_{i}}\right)}^{2}}{2(n-2)}$$

or more simply

$$\lambda_{2,\;2(n-2)}=\frac{nD^{2}}{2\sigma^{2}}$$

where *D* is the Euclidean distance between the centroids of the two treatment groups in 2-dimensional space. Extension to multiple dimensions is straightforward. If you further define

$$C=\frac{D}{\sigma }\;,$$

as the ratio of the distance between assemblages to the within-assemblage standard derivative, then

$$\lambda_{2,\;2(n-2)}=\frac{nC^{2}}{2}$$

The quotient *C* is an expression of the signal-to-noise ratio in the two-treatment comparison. The numerator of *C* is the linear distance between treatment centroids in any number of dimensions. The denominator of *C* is the standard deviation for the within-treatment, between-samples distances. The value *C* is a unitless measurement of the relative size of the treatment difference compared to the within-treatment standard deviation in distance. The unitless dimension of *C* is helpful when analyzing principal components that are inherently difficult to interpret in terms of units. The quotient *C* is a measure of how many standard deviations the treatment centroids are apart under the alternative hypothesis of heterogeneous communities. The larger *C* becomes, the easier it is to detect treatment differences for a fixed sample size *n*.

When there are three or more treatments, power calculations can still be based on the quotient *C*. However, *C* is expressed as

$$C^{'}=\frac{D_{MAX}}{\sigma }\;,$$ (3)

where the numerator is now the maximum expected distance between any two of the treatment groups in the study and σ is again the between-replicate, within-treatment standard deviation. The degrees of freedom in the noncentrality parameter, $\lambda_{{df}_{1},{\;df}_{2}}$, are now based on the degrees of freedom in the associated *F*-test with multiple treatments. Using *C’*, the minimum statistical power of the *F-*test is calculated assuming the remaining treatments have data centers equal to the grand centroid of the data. In any other treatment configuration, the power of the *F-*test will be greater than that specified by *C’*. When there is interest in the statistical power to compare two specific treatments, the earlier noncentrality parameter should be used (Eq (2)), with 2 and 2(*N*—*K*) degrees of freedom.

Using R software, the general form of the line commands for the power calculations are as follows:

$$crit.val<-\;qf\;((1-alpha),\;{df}_{1},\;{df}_{2})$$

$$1-pf\;(crit.val,\;{df}_{1},\;{df}_{2},\;ncp=\lambda )$$

The first line calculates the critical value under the null hypothesis for the *F*-test performed at an α-level = alpha, two-tailed, with degrees of freedom df_1_ and df_2_. The second line then calculates the statistical power (i.e.,1 –β) at the above critical value (crit.val) and degrees of freedom df_1_ and df_2_ when the noncentral parameter (ncp) is set to *λ*.

**Fig 6:**
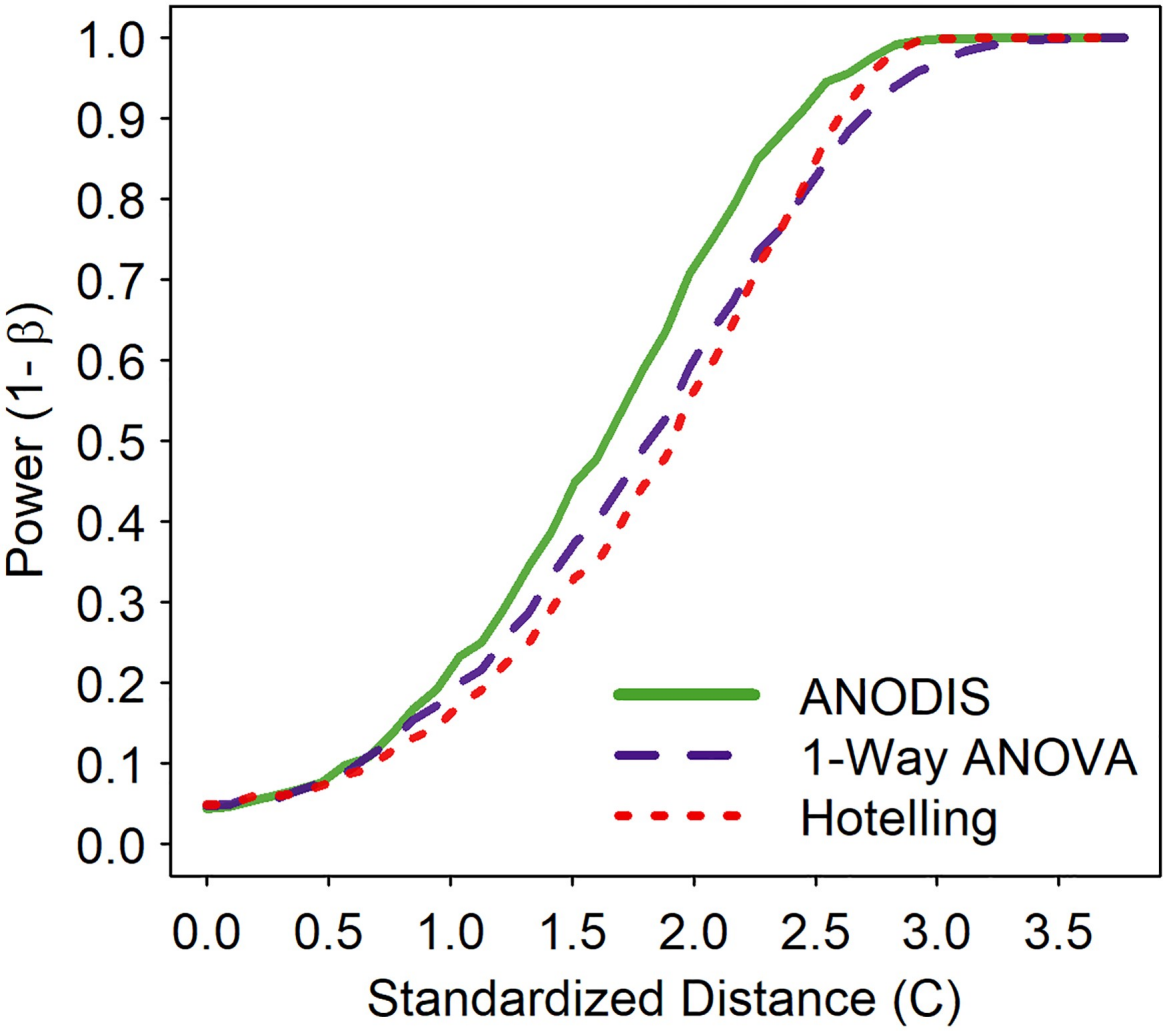
Example of simultaneous results under *H*_*α*_. Comparison of power curves for ANODIS, Hotelling’s T^2^ and separate ANOVA’s on individual PC’s as a function of standardized distance (i.e. *C*) between the two communities (K = 2) with r = 5 species per community assemblage and *n* = 5 samples per community at α = 0.05, 2-tailed. Other simulated scenarios produced similar results where ANODIS had statistical power greater than or equal to the other two data analysis approaches. Results illustrated for biplot PCA data.

Monte Carlo simulations of community treatment differences under *H*_*a*_ uniformly found the statistical power of the ANODIS to be greater than or equal to the alternative tests using Hotelling’s T^2^ or separate ANOVAs on the individual principle components. Hotelling’s T^2^ performed very similar to the individual ANOVA’s approach under all circumstances. The greatest difference in statistical power between ANODIS at the other two methods of analysis occurred when samples per community were small, i.e. *n* = 5 (Fig 6). The power of the three alternative methods asymptoted as sample sizes per community increased, i.e., *n* ˃ 20.

For convenience, sample size tables were constructed associated with the *F-*test in the ANODIS for the special case of a two-treatment randomized design with equal sample sizes (S2 Appendix). The statistical power of the *F-*test in the ANODIS is a function of both *C* and sample size per treatment group (i.e., *n*). The smaller the within-treatment sample size, the lower the statistical power of the test. Required per-treatment sample sizes (*n*) were calculated for statistical power of 1 –β = 0.70, 0.80, and 0.90 at α = 0.10, 0.05, and 0.01, two-tailed, for various values of *C*. The minimum sample size requirements are provided when simultaneously analyzing 1, 2, or 3 principal components. The sample sizes when analyzing one-dimensional data are an extension of tables provided by Kirk (pp. 840–841 [22]) for an *F-*test in the ANOVA for a two-treatment, completely randomized design.

## Results

### Dragonfly assemblages example

In the original analysis of the dragonfly assemblages by Magoba and Samways [11], it was concluded based on visual inspection that dragonfly assemblages did not differ between densely forested sites with alien trees and forests with natural trees (Fig 1) in upriver zones in South Africa. However, dragonfly assemblages at forested sites were visually concluded to differ from sites cleared of alien trees. ANODIS confirmed significant differences in dragonfly assemblages between cleared and forested sites (*P*(*F*_2,138_ ≥ 17.0948) < 0.0001). However, ANODIS also found forested sites invaded with alien trees to have significantly different dragonfly assemblages (*P*(F_2,88_ ≥ 4.3689) = 0.0155) than forested sites with only natural trees. For comparison, when using univariate ANOVAs on the separate principal components (0.0322 ≤ *P* ≤ 0.6007, α_TW_ = 0.0253 for two independent tests) and Hotelling’s *T*^2^ test (*P* = 0.1027) simultaneously on the two principal components, no significant differences were detected between alien and natural forested sites.

### Mammalian assemblages example

Using visual inspection of the bivariate principal component scatterplot of mammalian assemblages (Fig 2), Louys et al. [13] found that Central/South American communities were clearly differentiated from African and Asian communities along the first principal component, which explains 36% of the variation. The overall F-test from ANODIS was highly significant (P < 0.0001, Table 2). Construction of the ANODIS table and resulting F-tests are illustrated in Table 2. Orthogonal contrasts (S1 Appendix) were used to compare Central/South American vs African/Asian communities and compare African vs Asian communities. Calculations of contrast sums of squares is explained in S1 Appendix, and R code script (S5 File) to perform the calculations is provided for convenience.

**Table 2:** Construction of an ANODIS table using one-way ANOVA results for principle components PC1 and PC2 for mammal assemblage data from [13]. Linear contrast *C*_1_ compares central/South American communities to the average of African and Asian communities. Linear contrast *C*_2_ compares African vs Asian communities.

	*PC1 –ANOVA*		*PC2 –ANOVA*		ANODIS				
Source	DF	SS	DF	SS	DF	SS	MS	F	P
Total COR	61	4182.38	61	2926.96	122	7109.34			
Treatment	2	2856.60	2	487.89	4	3344.49	836.12	26.21	< 0.0001
*C*_1_	1	2454.71	1	13.60	2	2468.31	1234.16	38.68	< 0.0001
*C*_2_	1	277.40	1	483.02	2	760.42	380.21	11.92	< 0.0001
ERROR	59	1325.78	59	2439.07	118	3764.85	31.91		

ANODIS confirms the assemblage differences between Central/South American and African/Asian communities (*P*(*F*_2,118_ ≥ 38.68) < 0.0001). However, appreciable overlap between African and Asian communities makes visual interpretation difficult. Louys et al. (2011) conclude that African and Asian communities show very high convergence in structure based on visual inspection of the principal component biplot (Fig 2). In contrast, however, ANODIS finds differences between Asian and African mammalian community structures to be highly significant (*P*(*F*_2,118_ ≥ 11.92) < 0.0001). For comparison, both univariate analyses on the separate principal components (i.e. Asian vs. African 0.0010 ≤ *P* ≤ 0.0016) and Hotelling’s T^2^ test (i.e. Asian vs. African *P* < 0.0001) based on the two principal components simultaneously support the ANODIS results, but the *P*-values are generally higher.

### Stream chemistry example

In the analysis of differences in water chemistry between impacted and non-impacted sites with different underlying geology, the first three principal components explained 80% of variability in the dataset. Metals including Mn, Cu, Zn, and Ni, as well as sulphate loaded strongly onto the first principal component, while lead and organic carbon loaded strongly onto the second principal component. Annala et al. [14] visually conclude from the bivariate scatterplot of the first two principal components that acidic and neutral sites were distinguishable, but that pristine and impacted sites were almost completely intermixed. Consistent with the authors’ conclusions, ANODIS found a highly significant difference among acidic and neutral sites (*P*(*F*_2,82_ ≥ 28.8192) < 0.0001), but a lack of differentiation between impacted and near-pristine sites (*P*(*F*_2,82_ ≥ 1.8097) = 0.1702). For comparison, ANOVAs on the separate principal components (0.0001 ≤ *P* ≤ 0.0619, *α*_TW_ = 0.0253 for two independent tests) as well as Hotelling’s T^2^ (*P* < 0.0001) test found similar conclusions comparing acidic vs neutral sites. However, in the comparison of impact versus near pristine sites, *P*-values for the separate ANOVAs on the two principal components (0.3594 ≤ *P* ≤ 0.3939) and Hotelling’s T^2^ test (*P* = 0.4258) were also insignificant, but *P*-values were higher, indicative of the lower statistical power of these standard tests. ANODIS found no evidence of an interaction effect between underlying geology and impact status (*P*(*F*_2,82_ ≥ 0.4811) = 0.6198) on water chemistry.

### Power and sample size requirements

Sample size tables were constructed associated with the *F-*test in the ANODIS for the special case of a two-treatment randomized design with equal sample sizes (S1 Appendix). The provided sample sizes are a useful guide in the initial design of community analyses. All else being equal, the required sample sizes increase as the number of dimensions of the PCA to be analyzed increases. The implication is that more sampling effort is required to compare community structures than that required to simply compare the abundance of a single species across treatments. However, the increase in sample sizes needed for community analysis are relatively small, considering the potential gain in information. For a statistical power of 1 –β = 0.80, at α = 0.10, two tailed, and *C* = 1.0, sample sizes for one-dimensional population analysis are *n* = 14, while two- and three-dimensional community analyses have required sample sizes of *n* = 17 and 21, respectively.

As described earlier, *C* is a measure of the number of standard deviations the treatment centroids are apart. It is therefore convenient in power calculations to envision the size of the treatment difference in terms of this relative measure of distance. Statistical power increases as the value of *C* increases for all else being equal. In *post hoc* power calculations, the value of *C* should not be based on what original occurred, rather on the size of *C* considered important to detect, if it indeed occurred (22:143).

In the case of the stream chemistry study, the average sample size was *n* = 11. With four treatment groups, minimal statistical power can be calculated using *C'* (Eq (3)). The value of *C'* is the relative distance measured in the number of standard deviations between the two most exteme treatments with the remaining treatment centroids located in between. That study with per treatment sample size of *n* = 11 would have numerator degrees of freedom of

$${df}_{1}=D(K-1)=2(4-1)=6.$$

For a 2-dimensional (i.e., *D* = 2) bivariate scatterplot ANODIS, the denominator degrees of freedom would be

$${df}_{2}=D(\sum_{i=1}^{K}n_{i}-K)=2(44-4)=80.$$

If interest is in detecting a treatment difference of *C'* = 1.0 the noncentrality parameter is calculated to be

$$\lambda_{6,80}=\frac{n{\left(C^{'}\right)}^{2}}{2}=\frac{11∙{\left(1.0\right)}^{2}}{2}=5.5.$$

Setting *α* = 0.10, two-tailed, the statistical power is calculated to be 1 –β = 0.4733 using the R commands

crit.val<-qf((1-0.10),6,80)

1-pf(crit.val,6,80,5.5)

In the case of *C’* = 2.0, *λ* = 22.0 and the statistical power increases to 1 –β = 0.9732, at α = 0.10, two-tailed. R scripts (S6 File) to calculate *λ* and statistical power have been provided for convenience.

## Discussion

Reducing the subjective interpretation of bivariate scatterplots of PCA values should be helpful in ecological investigations. Based solely on visual inspection, treatment differences can be overlooked or perceived incorrectly based on individual perception. Reproducible, objective criteria in interpreting PCA bivariate scatterplot data should help eliminate one readily controllable source of uncertainty in community studies.

There are several assumptions inherent in ANODIS. The *F*-tests in the ANODIS are based on the assumptions the initial samples are independent and the principal components are orthogonal. If the original field survey collected independent samples, then the observations will remain independent, regardless of transformation (p. 104 [23]). By design, second- and higher-order axes are orthogonal (i.e., independent) to all previous axes in PCA [20] and CCA [24]. Seber [21] formally demonstrated that principal components are uncorrelated.

The *F*-tests in ANODIS also share the same assumptions as the *F*-tests in univariate ANOVA from which they were derived. The key assumptions are normally distributed data and constant variance. The robustness of ANOVA to these assumption violations has been thoroughly studied (see Zar, pp. 200–202 [25], for a review). Because ANODIS is a function of univariate ANOVAs, its robustness to these assumption violations can be directly inferred. If the original species data used in PCA are multivariate normal, then the individual principal components will be exactly univariate normal (p. 447 [26]). In the case where species composition data are expressed as proportions, then that multinomially distributed data will be asymptotically multivariate normal just as binomial data are commonly approximated by a univariate normal distribution (p. 533 [25]). In general, because the principal components are linear functions of the original data, by the central limit theorem (pp. 206–208 [27]), they will be asymptotically univariate normal. However, the property of normality cannot be assured for every application. Consequently, investigators should test this assumption of normality before using ANODIS. Normality can be visually assessed using quantile–quantile plots (pp. 87–88 [28]) or quantitatively tested using the D’Agostino–Pearson test (pp. 116–117 [28]). This chi-square test of normality combines with results from separate tests for skewness and Kurtosis and is considered to be more powerful than the more generic Kolmorgorov-Smirnov goodness-of-fit test (28:133–140) to a cumulative normal distribution. R code script for the D’Agostino-Pearson test is provided (S7 File). For the three examples used in this paper, the null hypothesis of normality was not rejected for any set of the principal components (α_EX_ = 0.05).

The adverse effects of nonnormality will generally be small if sample sizes are equal, or sample sizes are large, or when the underlying distributions of the principal components are symmetric but not normally distributed [29–31]. With small sample sizes, strong platykurtosis (i.e., flatter than normal) will decrease statistical power, while leptokurtosis (i.e., more peaked than normal) will increase statistical power [30].

As an ANOVA-based approach, ANODIS will generally be liberal if within-treatment variances are unequal [32]. Myers and Wells (p. 221 [33]) found the Type I error to increase by less than 0.02 when α was set at 0.05 and variances differed by less than a factor of 4. The analyses will be more robust to heterodasticity if sample sizes are equal. If the larger treatment sample size is associated with the larger variance, the tests will be conservative (i.e., Type I error will be less than α) and the *F*-test will be liberal (i.e., Type I error will be greater than α) when the larger variance is associated with the smaller sample size [34]. Statistical power will track these directional changes in Type I error. The consequence is that ANOVA, MANOVA, and ANODIS may reject the null hypothesis of same mean location when the actual effect is a change in dispersion. Warton et al. [35] found both the nature of species abundance information (e.g., abundance counts, proportions, etc.) and choice of statistical approach will potentially confound location and dispersion effects when analyzing community composition data.

In the process of reducing the dimensionality of community composition data, PCA imposes a linear relationship between predictors and response, which might be disadvantageous. If there is nonlinearity, PCA will represent the information in a higher number of principal components than necessary. Many ecologists prefer CCA and related ordinations to PCA because the underlying models assume unimodal responses rather than gradients.

Timm (pp. 449–457 [26]) summarizes several formal and informal approaches to determining the appropriate number of principal components to use in summarizing the multivariate data. Cattell [36] suggested creating a scree plot by graphing estimated eigenvalues against the number of principal components, then identifying the spline point where the change in consecutive eigenvalues diminishes with increasing number of dimensions. Generally, the selected principal components should account for 70–80% of the total variability (p. 450 [26]).

Alternatives to the use of ANODIS include both parametric and nonparametric approaches. Perhaps the simplest alternative is to perform separate univariate ANOVAs on each principal component. In this case, the test-wise (TW) α-level must be adjusted downward to control the experimental-wise (EX) error rate, i.e.,

$$1-{\left(1-\alpha_{TW}\right)}^{m}=\alpha_{EX}.$$

For example, with *m* = 2 principal components being compared, the test-wise alpha level must be set to α_TW_ = 0.0253 to retain α_EX_ = 0.05. With half the error degrees of freedom and a smaller α-level, the univariate ANOVA approach will have lower statistical power than ANODIS, as demonstrated by our Monte Carlo simulations. Hotelling’s T^2^ test in the case of two treatments (p. 99 [26]) or MANOVA in the case of multiple treatments will also halve the error degrees of freedom relative to ANODIS and, again, result in lower statistical power. Both the univariate and MANOVA alternatives share the same assumptions of normality and equal variances as ANODIS.

Instead of using the eigenvalues to compare treatments, Krzanowski [37] recommended using the eigenvectors to compare different treatment groups. The eigenvectors of each sample group are written as the columns of matrices L and M, respectively, for the two groups, then the “critical angles” between the two sets of principal components are obtained from the eigenvalues of the matrix LM′ML′. A limited set of critical values were generated by Krzanowski [16] at α = 0.05 to test the null hypothesis of equal principal component matrices but no sample size calculations were provided.

Nonparametric multivariate approaches to test for differences in community structure between treatment groups have become popular [38–40]. The approaches use permutated resampling of the data and base inferences on the empirically derived distribution of possible results from MANOVA. Consequently, the approaches may go by the names permutational MANOVA, nonparametric MANOVA, or PERMANOVA. These nonparametric methods relax the assumption of normality but still require the assumption of equal dispersion (e.g., equal variance) [41]. Unequal within-treatment dispersion may result in confounding differences in location with differences in dispersion which can also occur in parametric approaches. The gains in robustness are somewhat offset by lower statistical power if the data are indeed normally distributed. Although these procedures offer a nonparametric alternative, they are not without assumptions [41]. In particular, permutation tests should only be used when treatments were actually initially randomized to sites (pp. 627–628 [41]). More importantly, for purposes of study design and planning, permutation tests do not readily lend themselves to power calculations or sample size determination.

A singularly valuable contribution of this ANODIS approach and the associated *F-*test is the ability to readily perform sample size calculations. Too often in the past, community analyses have been conducted without regard to statistical performance, leaving little but anecdotal evidence and poorly supported conclusions. Proper design, data analysis, and sufficient sample sizes should help reverse this tendency.

## Supporting information

S1 Appendix S1_Appendix A.docx. Equation to test linear contrasts (L) in ANODIS.
(DOCX)

S2 Appendix S2_Appendix B.docx. Minimum samples sizes needed to ensure a given power 0.70, 0.80, and 0.90.
(DOCX)

S1 File S1_File Dragonfly assemblage data.csv. First two principal component values from dragonfly study.
(CSV)

S2 File S2_File Mammalian assemblage data.csv. First two principal component values from mammalian study.
(CSV)

S3 File S3_File Stream chemistry data.csv. First two principal component values from stream study.
(CSV)

S4 File S4_File anodis.txt. R script example to perform an analysis of distance.
(TXT)

S5 File S5_File SSc function.txt. R code function to estimate the Sum-of Squares with linear contrasts.
(TXT)

S6 File S6_File noncentrality r code.txt. R code function to estimate the noncentrality paramter in power calculations.
(TXT)

S7 File S7_File dagostino pearson test fn.txt. R code function to perform a test for normality.
(TXT)
