# Correction: A statistical test and sample size recommendations for comparing community composition following PCA

**DOI:** 10.1371/journal.pone.0257146

**Published:** 2021-09-01

**Authors:** John R. Skalski, Shelby M. Richins, Richard L. Townsend

There are errors in S4 File. The authors have provided a corrected version here.

## Supporting information

S4 File S4_File anodis.txt. R script example to perform an analysis of distance.
(TXT)
