# Supplementary material for: A statistical test and sample size recommendations for comparing community composition following PCA
Source: PLoS One. 2018 Oct 24;13(10):e0206033. doi: 10.1371/journal.pone.0206033 (PMC6200243; doi:10.1371/journal.pone.0206033)
Supplement: S1 Appendix — Equation to test linear contrasts (L) in ANODIS. (DOCX) [file pone.0206033.s001.docx]

**Appendix A**

To test linear contrasts (L) in ANODIS, the contrast sum of squares for each principle component is computed first as follows

$${SS}_{cx}=\frac{L^{2}}{\sum_{i=1}^{k} \left( \frac{c_{i}^{2}}{n_{i}} \right)}$$

where the linear contrast is defined as

L = $C_{1}\bar{x}_{1}$ + $C_{\alpha}\bar{x}_{2}$ + … + $C_{k}\bar{x}_{k}$

and where

$\bar{x}_{i}$ = mean for the first principle component for the *i*th treatment (*i*=1,…,K),

*C_i_* = coefficient associate with the *i*th treatment in the linear contrast.

The same linear contrast is calculated using the second principle component treatment means and an analogous contrast sum of squares calculated (i.e. SS_cy_). The contrast sum of squares in ANODIS is then the sum SS_cx_ + SS_cy_, with 2 degrees of freedom. The F-test for the contrast is then

$$F_{2,\mathrm{df}_{2}}=\frac{\left[ \frac{SS_{cx}+ SS_{cy}}{2} \right]}{\left[ \frac{\mathrm{SSE}}{2\left( \sum_{i=1}^{K} n_{i}-K \right)} \right]}$$
