# Supplementary material for: A statistical test and sample size recommendations for comparing community composition following PCA
Source: PLoS One. 2018 Oct 24;13(10):e0206033. doi: 10.1371/journal.pone.0206033 (PMC6200243; doi:10.1371/journal.pone.0206033)
Supplement: S2 Appendix — Minimum samples sizes needed to ensure a given power 0.70, 0.80, and 0.90. (DOCX) [file pone.0206033.s002.docx]

**Appendix B**

**Table B. Minimum sample sizes needed to ensure a given power** **a) 1 – β = 0.70, b) 1 – β = 0.80, and c) 1 – β = 0.90 to detect a relative treatment difference of size *C* at a significance level of α, two-tailed, for a two-treatment, randomized design.** Sample sizes are given when analyzing 1, 2, and 3 dimensions of a principal components analysis.

a. 1 – β = 0.70

| **Dimensions** | **α-level** | ***C*** | | | | | | | | |
| --- | --- | --- | --- | --- | --- | --- | --- | --- | --- | --- |
|  |  | **0.5** | **0.75** | **1** | **1.25** | **1.5** | **1.75** | **2** | **2.5** | **3** |
| **1** | 0.10 | 39 | 18 | 11 | 7 | 6 | 4 | 4 | 3 | 3 |
|  | 0.05 | 51 | 23 | 14 | 9 | 7 | 6 | 5 | 4 | 3 |
|  | 0.01 | 79 | 36 | 21 | 15 | 11 | 9 | 7 | 5 | 5 |
| **2** | 0.10 | 49 | 22 | 13 | 9 | 6 | 5 | 4 | 3 | 3 |
|  | 0.05 | 63 | 29 | 17 | 11 | 8 | 6 | 5 | 4 | 3 |
|  | 0.01 | 94 | 43 | 25 | 17 | 12 | 9 | 8 | 5 | 4 |
| **3** | 0.10 | 56 | 26 | 15 | 10 | 7 | 6 | 5 | 3 | 3 |
|  | 0.05 | 71 | 32 | 19 | 12 | 9 | 7 | 6 | 4 | 3 |
|  | 0.01 | 105 | 48 | 27 | 18 | 13 | 10 | 8 | 6 | 4 |

Table B (continued)

b. 1 – β = 0.80

| **Dimensions** | **α-level** | ***C*** | | | | | | | | |
| --- | --- | --- | --- | --- | --- | --- | --- | --- | --- | --- |
|  |  | **0.5** | **0.75** | **1** | **1.25** | **1.5** | **1.75** | **2** | **2.5** | **3** |
| **1** | 0.10 | 51 | 23 | 14 | 9 | 7 | 5 | 4 | 3 | 3 |
|  | 0.05 | 64 | 29 | 17 | 12 | 9 | 7 | 6 | 4 | 4 |
|  | 0.01 | 96 | 44 | 26 | 17 | 13 | 10 | 8 | 6 | 5 |
| **2** | 0.10 | 63 | 29 | 17 | 11 | 8 | 6 | 5 | 4 | 3 |
|  | 0.05 | 78 | 36 | 21 | 14 | 10 | 8 | 6 | 5 | 4 |
|  | 0.01 | 113 | 51 | 29 | 19 | 14 | 11 | 9 | 6 | 5 |
| **3** | 0.10 | 71 | 32 | 19 | 12 | 9 | 7 | 6 | 4 | 3 |
|  | 0.05 | 88 | 40 | 23 | 15 | 11 | 8 | 7 | 5 | 4 |
|  | 0.01 | 125 | 56 | 32 | 21 | 15 | 12 | 9 | 6 | 5 |

c. 1 – β = 0.90

| **t** | **α-level** | *C* | | | | | | | | |
| --- | --- | --- | --- | --- | --- | --- | --- | --- | --- | --- |
|  |  | **0.5** | **0.75** | **1** | **1.25** | **1.5** | **1.75** | **2** | **2.5** | **3** |
| **1** | 0.10 | 70 | 32 | 18 | 12 | 9 | 7 | 6 | 4 | 3 |
|  | 0.05 | 86 | 39 | 23 | 15 | 11 | 8 | 7 | 5 | 4 |
|  | 0.01 | 121 | 55 | 32 | 21 | 15 | 12 | 10 | 7 | 6 |
| **2** | 0.10 | 85 | 38 | 22 | 14 | 10 | 8 | 6 | 5 | 4 |
|  | 0.05 | 102 | 46 | 27 | 17 | 13 | 10 | 8 | 5 | 4 |
|  | 0.01 | 141 | 64 | 37 | 24 | 17 | 13 | 10 | 7 | 6 |
| **3** | 0.10 | 95 | 43 | 25 | 16 | 12 | 9 | 7 | 5 | 4 |
|  | 0.05 | 115 | 52 | 30 | 19 | 14 | 10 | 8 | 6 | 4 |
|  | 0.01 | 155 | 70 | 40 | 26 | 19 | 14 | 11 | 8 | 6 |
